# Identification of key neoculin residues responsible for the binding and activation of the sweet taste receptor

**DOI:** 10.1038/srep12947

**Published:** 2015-08-11

**Authors:** Taichi Koizumi, Tohru Terada, Ken-ichiro Nakajima, Masaki Kojima, Seizo Koshiba, Yoshitaka Matsumura, Kohei Kaneda, Tomiko Asakura, Akiko Shimizu-Ibuka, Keiko Abe, Takumi Misaka

**Affiliations:** 1 Department of Applied Biological Chemistry, Graduate School of Agricultural and Life Sciences, The University of Tokyo, Tokyo, Japan; 2 Department of Biotechnology and Agricultural Bioinformatics Research Unit, Graduate School of Agricultural and Life Sciences, The University of Tokyo, Tokyo, Japan; 3 School of Life Sciences, Tokyo University of Pharmacy and Life Sciences, Tokyo, Japan; 4 Laboratory for Biomolecular Structure and Dynamics, RIKEN Quantitative Biology Center (QBiC), Yokohama, Japan; 5 Tohoku Medical Megabank Organization, Tohoku University, Sendai, Japan; 6 Department of Physics, Kansai Medical University, Hirakata, Japan.; 7 Present Address: School of Life Sciences Tokyo University of Pharmacy and Life Sciences, Tokyo, Japan; 8 Niigata University of Pharmacy and Applied Life Sciences, Akiha-ku, Niigata, Japan; 9 Kanagawa Academy of Science and Technology, Takatsu-ku, Kawasaki-shi, Kanagawa, Japan

## Abstract

Neoculin (NCL) is a heterodimeric protein isolated from the edible fruit of *Curculigo latifolia*. It exerts a taste-modifying activity by converting sourness to sweetness. We previously demonstrated that NCL changes its action on the human sweet receptor hT1R2-hT1R3 from antagonism to agonism as the pH changes from neutral to acidic values, and that the histidine residues of NCL molecule play critical roles in this pH-dependent functional change. Here, we comprehensively screened key amino acid residues of NCL using nuclear magnetic resonance (NMR) spectroscopy and alanine scanning mutagenesis. We found that the mutations of Arg48, Tyr65, Val72 and Phe94 of NCL basic subunit increased or decreased both the antagonist and agonist activities. The mutations had only a slight effect on the pH-dependent functional change. These residues should determine the affinity of NCL for the receptor regardless of pH. Their locations were separated from the histidine residues responsible for the pH-dependent functional change in the tertiary structure. From these results, we concluded that NCL interacts with hT1R2-hT1R3 through a pH-independent affinity interface including the four residues and a pH-dependent activation interface including the histidine residues. Thus, the receptor activation is induced by local structural changes in the pH-dependent interface.

Humans recognise structurally diverse sweeteners, such as sugars, glycosides, d-amino acids, peptides, and proteins. All of these compounds activate the human sweet receptor hT1R2-hT1R3^1^,^2^. The majority of sweet molecules are of low molecular weight. However, eight proteins are known to elicit a sweet taste of their own, namely, brazzein^3^, lysozyme^4^,^5^, mabinlin^6^, monellin^7^, pentadin^8^, thaumatin^9^, miraculin^10^, and neoculin^11^,^12^. Some of these sweet proteins have been proposed to bind to specific sites on the receptor^13^,^14^,^15^,^16^,^17^,^18^. However, few common features in their primary and tertiary structures have been identified^19^. Therefore, how the human sweet receptor commonly receives these sweet proteins remains unclear.

Neoculin (NCL) is a sweet protein isolated from the edible fruit of the tropical plant *Curculigo latifolia*. NCL exhibits a taste-modifying activity that converts sourness to sweetness. NCL elicits a slightly sweet taste on its own and elicits a strongly sweet taste in the presence of an acidic solution^20^. This protein is a heterodimer composed of an acidic subunit (neoculin acidic subunit, NAS) of 113 amino acid residues and a basic subunit (neoculin basic subunit, NBS) of 114 amino acid residues. Although the two subunits share 77% amino acid identity, they differ in terms of their isoelectric points; the pI for NAS is between pH 4.0 and 6.0, whereas that for NBS is between pH 7.5 and 9.5^11^. They form a clamshell-like structure and are connected by two disulphide bonds (Fig. 1). We previously revealed that NCL acted as an hT1R2-hT1R3 antagonist at neutral pH and as an agonist at acidic pH; histidine residues were responsible for this pH-dependent functional change^21^. Of two and three NAS and NBS histidine residues, we demonstrated that NBS His11 functioned as a primary pH sensor^22^. Therefore, we inferred that protonation of the histidine residues caused a conformational change in NCL and influenced its binding to the receptor^21^.

In this study, we used NMR to comprehensively screen the NCL residues responsible for the pH-dependent functional change under various pH conditions. Then, we produced a series of NCL mutants to verify their contributions to the functional change. Based on the results obtained from these experiments, we have updated our model of how pH change affects the NCL function.

## Results

### NMR resonance assignments and ^1^H-^15^N chemical shift changes following pH titration

To screen residues whose conformations are influenced by the pH change, we measured NMR spectra for NCL at different pH values ranging from 3 to 7. For this experiment, we produced samples of uniformly ^13^C/^15^N-labelled NAS in complex with unlabelled NBS and uniformly ^13^C/^15^N-labelled NBS in complex with unlabelled NAS. Sequence- and subunit-specific NMR resonance assignments were made for the backbone ^1^HN, ^15^N and ^13^Cα and side-chain ^13^Cβ nuclei of most of the residues at pH 3 and 4 using standard triple-resonance NMR spectra^23^ (Supplementary Fig. S1 and Supplementary Table S1).

**Figure 2:**
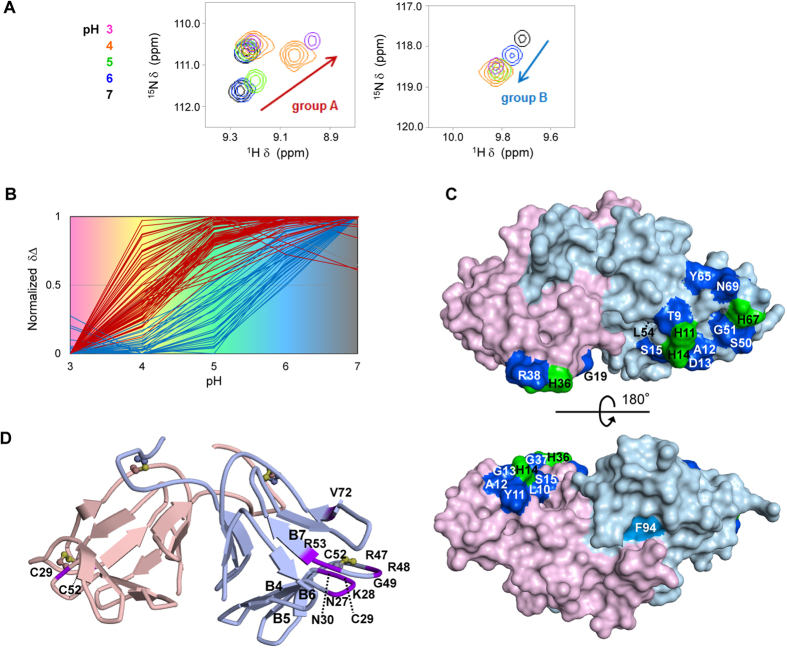
NMR-spectroscopy-based screening of the residues most likely to undergo conformational changes. (**A**) Close-up views of the superposition of NCL ^1^H-^15^N HSQC spectra recorded during the pH-titration experiment. See Supplementary Fig. S2 for all regions. pH of each spectrum is indicated by the colour scale. (**B**) Classification of all NCL residues that showed significant chemical shift changes between pH 3 and 7. All the residues are classified as two groups: the residues that showed chemical shift changes primarily between pH 3 and 5 are classified as “group A”, whereas those that showed chemical shift changes primarily between pH 5 and 7 are classified as “group B”. The residues classified as “group A” are coloured in red, whereas those classified as “group B” are coloured in blue. The residues classified as neither group A nor group B are coloured in grey. (**C**) Mapping of the residues classified as “group B” on the protein surface of the crystal structure of NCL at pH 7.4 (PDB ID: 2D04). All of these residues (coloured in blue) were located near His residues (coloured in green). NBS Phe94 (coloured in cyan) was specially selected as the target of the subsequent alanine-scanning mutagenesis because it exhibited a significant chemical shift change in the pH range of 5–7, although it was classified as group A. The overall structure of NCL is shown in two orientations separated by 180°. The protein surfaces of NAS and NBS are coloured in pale red and pale blue, respectively. (**D**) Mapping of the residues whose signals were missing in the NMR spectra on the diagram showing the three-dimensional backbone of NCL (coloured in purple). The cysteine residues forming disulphide bonds are shown as ball-and-stick models. Some β-strands are labelled according to our previous paper (Shimizu-Ibuka *et al.*,^25^). The backbone of NAS and NBS are coloured in pale red and pale blue, respectively.

Next, we measured ^1^H-^15^N HSQC NMR spectra at pH 5, 6, and 7 (Fig. 2A and Supplementary Fig. S2A-B). Backbone ^1^HN and ^15^N resonances of these spectra were assigned by successively extrapolating the trend of the chemical shift changes to a higher pH. We observed 36 and 33 residues of NAS and NBS, respectively, that exhibited significant differences of more than 0.05 ppm in the ^1^H chemical shift or 0.5 ppm in the ^15^N chemical shift between the spectra at pH 3 and 7 (Supplementary Fig. S2C-F). Plotting the normalised chemical shift changes against pH demonstrated that the residues could be classified into two groups (Fig. 2A,B): in group A, most of the chemical shift changes occurred between pH 3 and 5, whereas in group B, most of the chemical shift changes occurred between pH 5 and 7. Because the change from an antagonist to an agonist of NCL occurs between neutral and weakly acidic pH, the residues of group B are more closely related to this function. Because all of these residues are located near His residues in the tertiary structure of NCL (Fig. 2C), the conformational change likely occur upon protonation of the His residues. Moreover, although Phe94 of NBS was classified as group A, it exhibited a significant chemical shift change in the pH range of 5–7. This result suggests that Phe94 is also related to this function.

The process of NMR resonance assignments of NCL indicated that the signals for Cys29 and Cys52 of NAS and Asn27, Lys28, Cys29, Asn30, Arg47, Arg48, Gly49, Cys52, Arg53, and Val72 of NBS were missing from the NMR spectra, presumably due to conformational exchange on the millisecond time scale. In the previous NMR study using the NBS homodimer, the resonance of Cys29, Asn30, Arg47, Cys52, Arg53 and Val72 remained unassigned^24^. Of these residues, residues 27–30 and 47–49 are located in the loops between β4 and β5 and between β6 and β7, respectively (Fig. 2D). Because residues 28, 47, and 48 are not conserved between NAS and NBS (Asn28, Gly47, and Gln48 in NAS), they make the two loops of NBS more flexible than those of NAS. Because Val72 is close to Arg47 in the tertiary structure of NCL, it may also undergo conformational exchange together with the loops.

### Alanine scanning mutagenesis of the residues exhibiting conformational changes

To verify the contributions of the selected residues on NCL’s pH-dependent activity, we produced a series of NCL mutants and evaluated their sweetness. Fourteen residues were selected for alanine scanning. Thr9, Leu54, and Tyr65 of NBS were selected from group B due to their large chemical shift changes in the pH range of 5–7. Phe94 of NBS was also selected for the reason described above. The positions of these residues are shown in Fig. 2C. Asn27, Asp46, Arg47, Arg48, Asp66, and Val72 of NBS were selected from the residues in the vicinity of and within the flexible loops between β4 and β5 and between β6 and β7 (see Fig. 2D). His36 of NAS and His11 and His14 of NBS were selected for comparison because they were shown to be responsible in whole or in part for the pH-dependent functional change of NCL^22^. Tyr21 of NAS was selected because it is located on the plane formed by NAS His36, NBS His11, and NBS His14 in the tertiary structure of NCL, suggesting its involvement in the interaction with the receptor when the histidine residues directly interact with the receptor. CD spectra demonstrated that the replacement of these residues with alanine had no substantial effects on the secondary structures (Supplementary Fig. S3).

**Figure 3:**
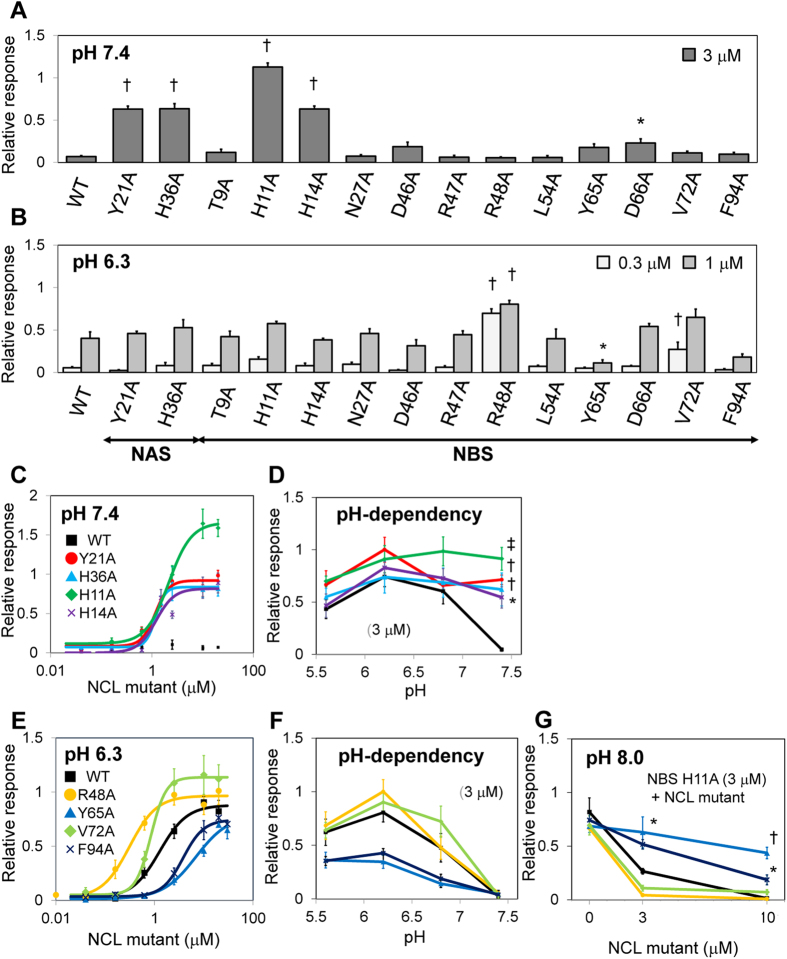
Production of NCL mutants and evaluation of their sweetness using the cell-based assay. (**A**,**B**) Responses of cells expressing hT1R2-hT1R3 and G15Gi3 to fourteen NCL mutants under neutral (pH 7.4) and weakly acidic (pH 6.3) conditions. The number of responsive cells was normalised relative to the maximum response to aspartame (6.7 mM) at pH 7.4. Error bars represent the mean ± SE (n = 4–6). *P < 0.05, †*P* < 0.001 vs. WT (one-way ANOVA followed by Dunnett’s test). (**C**–**F**) Dose-response relationships and pH dependencies of each set of four NCL mutants. The number of responsive cells was normalised relative to the maximum response to aspartame (6.7 mM) at pH 7.4. Each point represents the mean ± SE (n = 3–5). *P < 0.05, †*P* < 0.01, ‡*P* < 0.001 vs. WT (one-way ANOVA followed by Dunnett’s test). (**G**) Evaluation of the antagonistic activities of the four NCL mutants at pH 8.0. Each mutant was applied to cells expressing hT1R2-hT1R3 together with the NBS H11A mutant at pH 8.0. The number of responsive cells was normalised relative to the maximum response to aspartame (6.7 mM) at pH 8.0. Each point represents the mean ± SE (n = 4-5). *P < 0.05, †*P* < 0.001 vs. WT (one-way ANOVA followed by Dunnett’s test).

We evaluated the sweetness of each single-point NCL mutant at weakly acidic and neutral pH using the cell-based assay system established previously^21^. The human sweet receptor hT1R2-hT1R3 was transiently transfected into HEK293T cells together with G15Gi3 as a chimeric Gα. NCL or its mutant was applied to the cells under neutral (pH 7.4) or weakly acidic (pH 6.3) conditions (Fig. 3A,B). Consistent with the previous results^22^, replacement of His36 of NAS and His11 and His14 of NBS increased the receptor response at pH 7.4 (Fig. 3C) (the EC_50_ values for the mutants were: H36A, 1.20 μM; H11A, 2.12 μM; and H14A, 1.20 μM). The activity of the NBS H11A mutant was entirely independent of pH, whereas the activities of the NAS H36A and NBS H14A mutants were slightly lower at neutral pH compared with weakly acidic pH (Fig. 3D). Replacement of Tyr21of NAS also increased the response at neutral pH (EC_50_ value was 1.03 μM), although the response was slightly lower compared with weakly acidic pH. This result suggests that NAS Tyr21 also contributes to the pH-dependent functional change of NCL. Replacement of Arg48 and Val72 of NBS increased the response compared to the WT at pH 6.3, whereas replacement of Tyr65 and Phe94 of NBS decreased the response (Fig. 3E) (the EC_50_ values for the WT and mutants were: WT, 1.24 μM; R48A, 0.31 μM; V72A, 0.84 μM; Y65A, 6.90 μM; and F94A, 3.79 μM). Importantly, these changes had only a slight effect on the pH dependency of the response (Fig. 3F), in contrast to the mutations of NAS Tyr21 and His36 and NBS His11 and His14 (Fig. 3D). Next, we evaluated the antagonistic activity of the NBS R48A, Y65A, V72A, and F94A mutants under weakly basic conditions. Each mutant protein was applied to cells expressing hT1R2-hT1R3 together with the NBS H11A mutant at pH 8.0. Interestingly, replacement of Arg48 and Val72 of NBS strongly decreased the cell response induced by NBS H11A compared to the WT, whereas replacement of Tyr65 and Phe94 of NBS weakly decreased the response (Fig. 3G). These results suggest that these four residues in NBS contribute to both the agonist and antagonist potencies without affecting pH-dependency.

### Evaluation of the sweetness of the NCL double mutants

**Figure 4:**
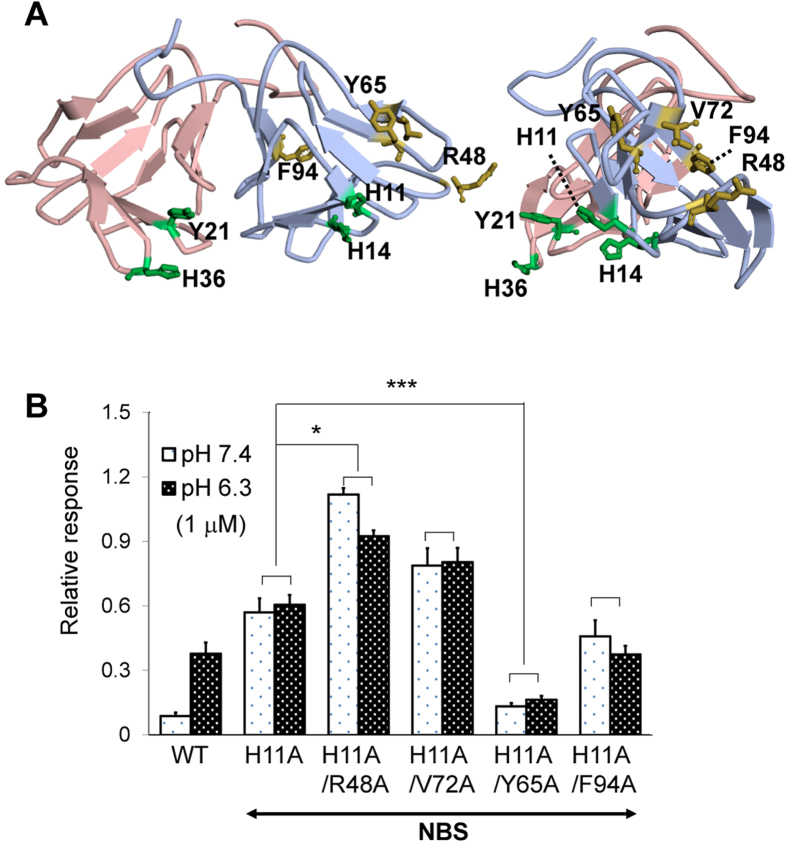
Evaluation of the sweetness of the NCL double mutants. (**A**) Separate locations of the residues responsible for the pH-dependency (green) or the agonist and antagonist potencies (yellow) are indicated on the diagram showing the three-dimensional backbone of NCL. The backbones of NAS and NBS are coloured in pale red and pale blue, respectively. The right diagram depicts the lateral side of NBS. (**B**) Cell-based analysis of the sweetness of the NCL double mutants. The responses of cells expressing hT1R2-hT1R3 and G15Gi3 were examined after the application of NCL double mutants carrying one substitution from each group of residues. All four double mutants showed equivalent activation of the cells under neutral (pH 7.4) and weakly acidic (pH 6.3) conditions. Additionally, their activation potencies were increased or decreased compared to the NBS H11A single-point mutant depending on the nature of the other mutation. The number of responsive cells was normalised relative to the maximum response to aspartame (6.7 mM) at pH 7.4. Error bars represent the mean ± SE (n = 4). *P < 0.05, ***P < 0.001 vs. NBS H11A single-point mutant (one-way ANOVA followed by Tukey’s *post hoc* test).

Figure 4A shows the distribution of the residues that contribute to NCL’s activity. The residues important for pH-dependency (NAS Tyr21 and His36 and NBS His11 and His14) are located separately from the residues important for both the agonist and antagonist potencies (NBS Arg48, Tyr65, Val72, and Phe94) in the tertiary structure. This result prompted us to evaluate whether the residues of these two groups function individually or cooperatively. For this purpose, four double mutants carrying one substitution from each group were produced. NBS His11 was substituted with Ala in all of the double mutants, whereas one of the Arg48, Tyr65, Val72, and Phe94 residues of NBS was substituted with Ala in each double mutant. Each double mutant protein was applied to cells expressing hT1R2-hT1R3 together with G15Gi3 under neutral (pH 7.4) or weakly acidic (pH 6.3) conditions. As shown in Fig. 4B, all four double mutants showed equivalent activation of the receptor at neutral and weakly acidic pH due to the loss of the pH-dependency regulated by NBS His11. Additionally, the activation level was increased compared to the NBS H11A mutant for the H11A/R48A and H11A/V72A double mutants and was decreased for the H11A/Y65A and H11A/F94A double mutants. Importantly, these tendencies are the same as those observed in the experiment using the single-point mutants (Fig. 3E). Therefore, the effects of the two groups on NCL’s activity are additive, suggesting that the residues of the two groups function individually.

## Discussion

In this study, we have newly identified the four NCL residues, Arg48, Tyr65, Val72, and Phe94 of NBS, responsible for both the agonist and antagonist potencies under acidic and neutral pH conditions based on NMR analysis and subsequent mutagenesis (Table 1). The EC_50_ values of the single-point alanine mutants of these residues for the receptor activation at pH 6.3 were shifted from that of WT. Furthermore, the antagonist potency at pH 8.0 was altered by the mutations in the same manner as the agonist potency was altered at pH 6.3. The results suggest that these residues determine the affinity of NCL for the receptor regardless of pH. In other words, they interact with the human sweet receptor, but this interaction is not largely influenced by the pH change. Although we assumed that Tyr65 and Phe94 of NBS exhibited pH-dependent conformational changes based on the NMR analysis, their pH-dependent conformational changes had only a slight influence, if any, on the interaction with the receptor.

**Figure 5:**
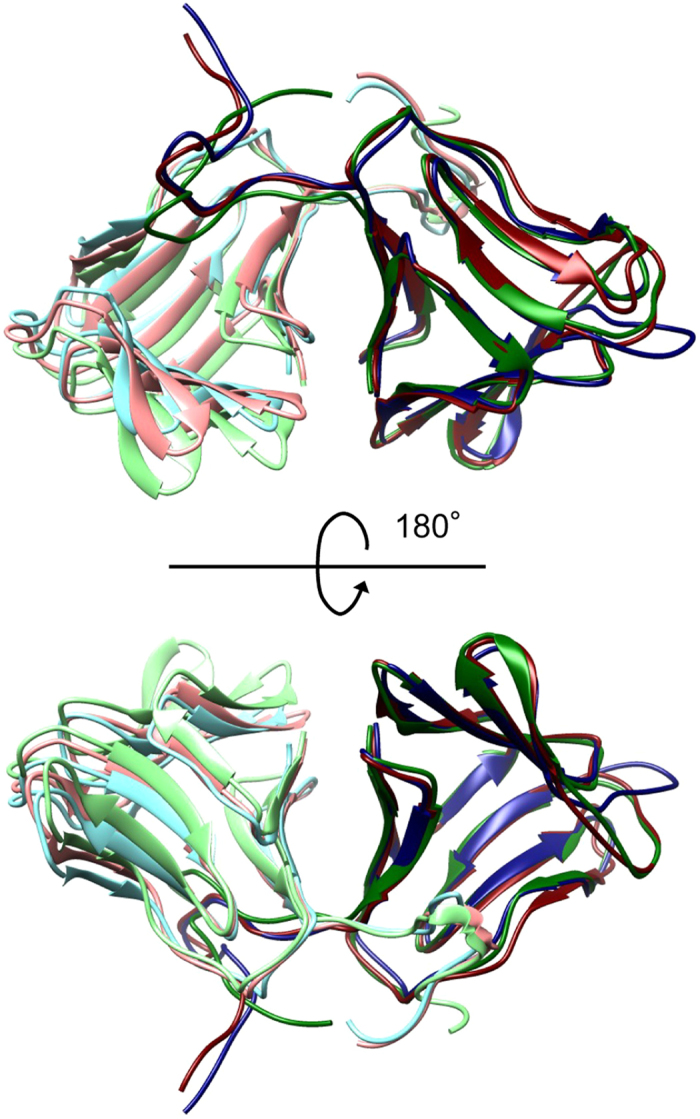
Comparison of the representative structures of the MD simulations under weakly acidic and neutral pH conditions with the crystal structure at pH 7.4 (PDB ID: 2D04). Structures are aligned with respect to NBS. NAS and NBS of the MD structure at pH 5, the MD structure at pH 7, and the crystal structure are coloured light red, dark red, light green, dark green, light blue, and dark blue, respectively. The structural images were generated using UCSF Chimera^39^.

**Figure 6:**
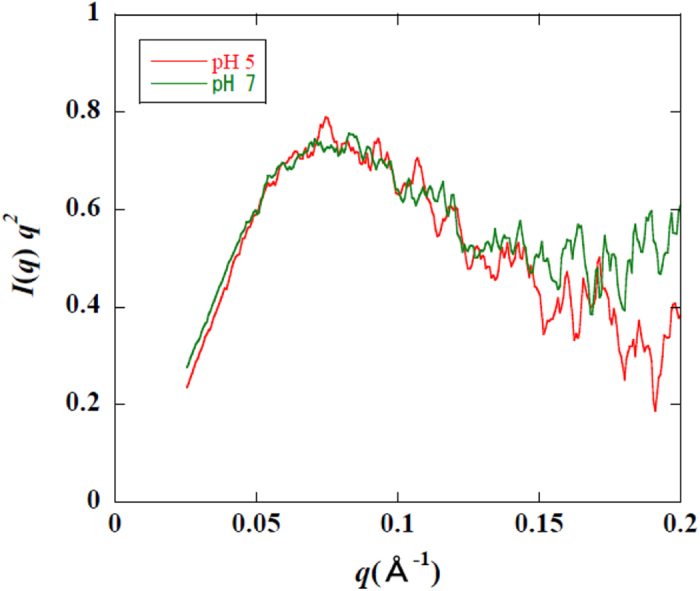
SAXS profiles of NCL at pH 5 and pH 7. The data are presented as Kratky plots, where 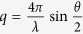 (*λ*, wavelength of irradiated X-ray; *θ*, scattering angle) and *I*(*q*) is the SAXS intensity at *q*. The lines at pH 5 and pH 7 are presented in red and green, respectively. The data at *q* < 0.025 are omitted because of the influence of the beam-stop of direct beam.

We previously hypothesised that NCL changes its conformation from closed to open as the pH decreases^25^. This hypothesis was based on the results of molecular dynamics (MD) simulations performed on NCL under neutral and acidic pH conditions. In the open conformation, the two subunits (NAS and NBS) comprising NCL were expected to partially dissociate from each other. However, the residues located in the interface between the two subunits did not show significant chemical shift changes in our NMR analysis between pH 5 and 7 (Fig. 2C). Moreover, only a limited number of residues showed significant chemical shift changes at weakly acidic pH in the entire NCL structure. The electrostatic repulsive interactions between the subunits may have been overestimated in the previous MD simulations because they used an implicit solvent model to reduce the computational cost. Therefore, we performed MD simulations for NCL under neutral and weakly acidic conditions with an explicit water model for an extended period of time (2 μs). Large conformational changes were not observed during the simulations, except for the loop between β6 and β7 of NBS (Fig. 5). The conformation of this loop significantly deviated from the conformation in the crystal structure in both simulations, which is consistent with the finding that the NMR signals of the residues in this loop were missing due to conformational exchange regardless of pH. Furthermore, SAXS analysis using NCL under neutral and acidic pH conditions indicated no difference in molecular size (Fig. 6). Therefore, NCL undergoes only local conformational changes at weakly acidic pH. Because this local change activates the receptor, the residues responsible for the local conformational change and pH-dependency should be located close to the receptor in the complex structure.

**Figure 7:**
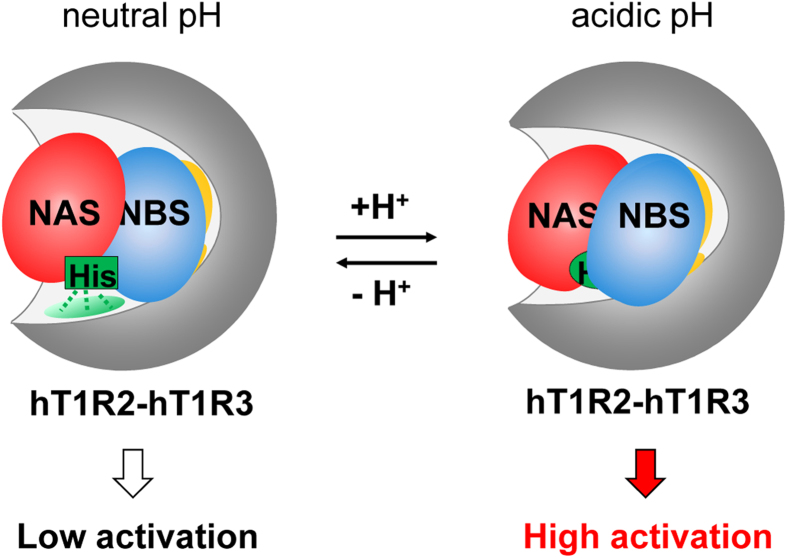
Probable model for the taste-modifying activity of NCL. Cartoon representation of the possible model. At neutral pH, NCL binds to the human sweet receptor and slightly activates it (*Left*). At acidic pH, the binding mode is locally altered by protonation of the histidine residues of NCL, leading to the strong activation of the receptor (*Right*).

The residues important for the pH-independent affinity were located separately from the residues responsible for the pH-dependency in the tertiary structure. Therefore, NCL interacts with the receptor through two interfaces: (1) the pH-independent affinity interface, whose interaction with the receptor is not influenced by the pH change and determines the affinity for the receptor, and (2) the pH-dependent activation interface, whose interaction with the receptor is altered by the pH change and induces the activation/inactivation of the receptor. In the pH-dependent activation interface, hydrogen bonds between the histidine residues of NCL and the receptor atoms suppress the activation of the receptor at neutral pH. Protonation of the histidine residues disrupts the hydrogen bonds and locally alters the structure of the interface, leading to the activation of the receptor at acidic pH (Fig. 7).

Thus, we updated our working hypothesis of the mechanism by which NCL exerts its taste-modifying activity. We propose that the local conformational change in the pH-dependent interface plays an essential role in activating the receptor. Identification of the receptor residues forming the pH-dependent interface should provide a clue to aid in the understanding of the mechanism of receptor activation. Furthermore, this type of study will provide new insight into the activation/inhibition mechanisms of other class C GPCRs.

## Methods

### Sweet molecules and solutions

Recombinant NCL was produced using a bacterial expression system^12^. The other sweeteners were purchased from commercial sources: aspartame and thaumatin from Wako Chemical Co, Ltd., Japan; sucralose from Tokyo Kasei Kogyo Co, Ltd., Japan; and cyclamate from Sigma Aldrich, USA.

### Preparation of NCL mutants

NCL mutants were produced using a bacterial expression system as mentioned above^12^. Briefly, cDNAs encoding NAS mutants or NBS mutants were constructed by PCR using overlapping primers that incorporated the mutations of interest. The pET-21b vector carrying NAS or NBS was used as a template for mutagenesis. Each cDNA was introduced into a pET21b expression plasmid at the *Sca*I and *Sph*I sites. Each expression plasmid was transformed into *Escherichia coli* BL21 Codon Plus (DE3) RIL cells. Expression and purification of the NCL mutant heterodimers were performed as described previously^22^.

### NMR data acquisition and assignments

For the NMR measurements, we prepared a wild-type NCL protein in which only one subunit was uniformly labelled with stable isotopes. The ^13^C/^15^N-labelled wild-type NAS and NBS subunits were expressed individually using the same bacterial expression system as described previously^12^ with M9 medium containing [U-^13^C] glucose and ^15^NH_4_Cl as the sole carbon and nitrogen sources, respectively. Similarly, the ^15^N-labelled subunits were expressed using M9 medium containing ^15^NH_4_Cl as the sole nitrogen source. Then, heterodimers of the selectively labelled subunits were produced by refolding the isotopically labelled subunits in the presence of their unlabelled counterparts as described previously^12^.

The protein was dissolved in NMR buffers (20 mM sodium citrate, pH 3–7 and 90% H_2_O/10% D_2_O) at concentrations ranging from 0.03 mM (pH 7) to 0.3 mM (pH 3). NCL was less soluble at the higher pH. The NMR experiments were conducted at 37°C with a sample volume of 300 μl in 5 mm tubes (Shigemi Co, Ltd., Japan) using a Bruker Avance 700-MHz spectrometer (Bruker, Germany) equipped with cryogenic probes. ^1^H-^15^N HSQC, HNCA, HN(CO)CA, CBCA(CO)NH, and HNCACB spectra were acquired at pH 3.0 and 4.0 using the ^13^C/^15^N-labelled samples. All spectra were processed using Azara-2.7 (www2.ccpn.ac.uk/azara) and were analysed using CcpNmr analysis^26^. The backbone ^1^HN, ^15^N and ^13^Cα and side-chain ^13^Cβ resonances were sequence- and subunit-specifically assigned using the triple resonance NMR spectra. The chemical shifts of these nuclei in NCL are listed in Supplementary Table S1.

### Cell culture and calcium imaging

HEK293T cells were kindly provided by Dr. Hiroaki Matsunami (Duke University). Cell cultures and transfections were performed as described previously^21^ but with a slight modification in the transfection procedure. hT1R2, hT1R3, G15Gi3 were transiently transfected into HEK293T cells at a ratio of 58:10:10 using the Lipofectamine 2000 reagent (Invitrogen, USA). Calcium imaging was performed as described previously^21^. The pH values of the tastant solutions were preadjusted by the addition of 20 mM citric acid. The number of responding cells was normalised relative to the response to 6.7 mM aspartame at pH 7.4. Nonspecific responses induced by solely pH changes were often observed below pH 5.5.

### Molecular dynamics simulations

Molecular dynamics simulations were performed for the wild-type NCL under neutral and weakly acidic conditions. The coordinates of chains C (NAS) and D (NBS) of the crystal structure of NCL (PDB ID: 2D04) were used to generate the initial structure of each simulation. Because the C-terminal residues (residues 112–114) of chain D were missing, they were added in an extended conformation. For the simulation under weakly acidic condition, histidine residues were protonated on both the Nδ1 and Nε2 atoms, whereas they were protonated on only the Nε2 atoms for the simulations under neutral conditions. The generated protein structures were immersed in water boxes of approximately 80 × 90 × 90 Å. Sodium and chloride ions were added to the boxes at concentration of approximately 30 mM using the solvate 1.0 program and the LEaP module of AmberTools 12^27^ to ensure that the net charges of the entire system were zero. Amber ff99SB force field^28^ and the TIP3P model^29^ were used for the proteins and water molecules, respectively.

Each system was subjected to energy minimisation and was equilibrated in the constant-NPT ensemble at 300 K and 1.0 × 10^5^ Pa for 1 ns. Then, a production run was performed for each system in the constant-NPT ensemble for 2 μs. Temperature and pressure were controlled using the velocity rescaling method^30^ and weak coupling method^31^, respectively. Electrostatic interactions were calculated using the particle mesh Ewald method^32^,^33^. The lengths of the bonds involving hydrogen atoms were constrained with the LINCS algorithm^34^,^35^ to allow for the use of a large time step (2 fs). The coordinates were recorded every 10 ps. The simulations were performed using GROMACS 4.5.5^36^.

### SAXS measurements and analyses

Small-angle X-ray scattering (SAXS) measurements were performed on beamline 15A of the Photon Factory at the High Energy Accelerator Research Organization, Tsukuba, Japan. The sample solution in a mica cuvette with 1 mm path length was irradiated with a monochromatic X-ray beam (1.5 A). All the data were obtained with CCD-based X-ray detector (Hamamatsu Photonics K.K., Japan), and were corrected for distortion of images, non-uniformity of sensitivity and contrast reduction for an X-ray image intensifier^37^. Scattering data in different solutions were corrected for the attenuation of the incident X-ray flux.

The exposure time was 24 s in one measurement, and the total of nine scans were accumulated with a sample in cuvette exchanged every three times. The protein concentration of the sample was 1 mg/mL, 0.5 mg/mL, and 0.25 mg/mL, and its dependency was checked before analyses. The radius of gyration was determined using Guinier approximation^38^.

### Statistical data analysis

The sweetness of each single-point NCL mutant (Fig. 3A–F) and the antagonistic activity of the mutants at weakly basic pH (Fig. 3G) using the cell-based assay system were tested for statistical significance using one-way ANOVA followed by Dunnett’s test. The sweetness of the NCL double mutants with the NBS H11A single-point mutant (Fig. 4B) was tested for statistical significance using one-way ANOVA followed by Tukey’s *post hoc* test.

## Additional Information

**How to cite this article**: Koizumi, T. *et al.* Identification of key neoculin residues responsible for the binding and activation of the sweet taste receptor. *Sci. Rep.* **5**, 12947; doi: 10.1038/srep12947 (2015).

## Supplementary Material

Supplementary Information

Supplementary Table S1

## Figures and Tables

**Figure 1:**
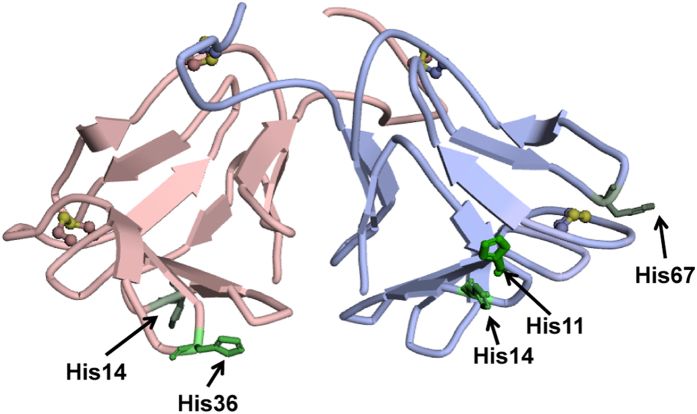
Crystal structure of NCL at pH 7.4 (PDB ID: 2D04). Neoculin is a heterodimeric protein composed of the neoculin acidic subunit (NAS) and neoculin basic subunit (NBS). NAS is indicated with a pale red ribbon, whereas NBS is indicated with a pale blue ribbon. The cysteine residues forming disulphide bonds are represented as ball-and-stick models and coloured in yellow. The histidine residues are shown with a stick model. NBS His11, a primary pH sensor of NCL, is coloured in green, whereas NAS His36 and NBS His14, which are partially responsible for the pH-dependency of NCL, are coloured in pale green. All structural images (with the exception of Fig. 5) were generated using Discovery Studio Visualizer 3.0 (Accelrys Software Inc).

**Table 1:** Effects of the ala substitution.

	pH sensitivity	agonistic activity (pH 6.3)	antagonistic activity (pH 8.0)
Tyr21 (NAS)	↓	—	n.d.
His36 (NAS)	↓	—	n.d.
His11 (NBS)	↓	—	n.d.
His14 (NBS)	↓	—	n.d.
Arg48 (NBS)	—	↑	↑
Tyr65 (NBS)	—	↓	↓
Val72 (NBS)	—	↑	↑
Phe94 (NBS)	—	↓	↓
