# Supplementary material for: Identification of key neoculin residues responsible for the binding and activation of the sweet taste receptor
Source: Sci Rep. 2015 Aug 11;5:12947. doi: 10.1038/srep12947 (PMC4542694; doi:10.1038/srep12947)
Supplement: Supplementary Information [file srep12947-s1.pdf]

Supplementary information

Identification of key neoculin residues responsible for the binding and activation of the sweet taste receptor

Taichi Koizumi, Tohru Terada, Ken-ichiro Nakajima, Masaki Kojima, Seizo Koshiba, Yoshitaka Matsumura, Kohei Kaneda, Tomiko Asakura, Akiko Shimizu-Ibuka, Keiko Abe, and Takumi Misaka

Supplementary methods

Far-UV CD spectra. Neoculin or its mutant was dissolved in 20 mM sodium phosphate buffer, pH 7.0 or 20 mM sodium citrate buffer, pH 3.0. Each solution was prepared at a protein concentration of 4 μM. Far-UV CD spectra were recorded in a 1 mm cell at 25°C using a J-820 spectropolarimeter (Jasco, Tokyo, Japan). All spectra were corrected by subtraction of the spectrum obtained using the buffer alone. The results from four scans were averaged.

Supplementary figure legends

Table S1 Chemical shift assignments of the neoculin acidic subunit (A) and neoculin basic subunit (B) at 37°C and pH 3.0.

A

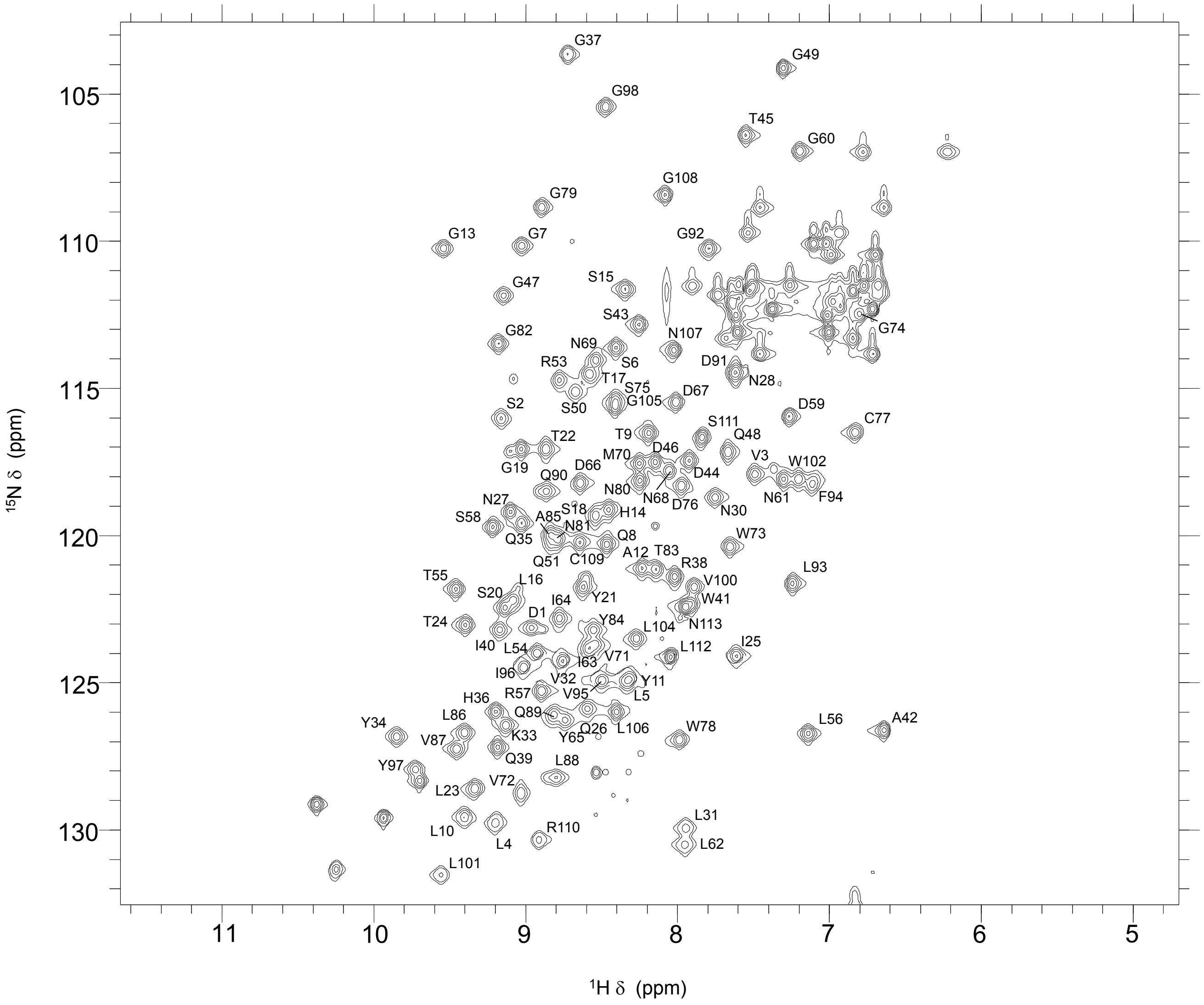

Figure S1 A ^{1}H-^{15}N HSQC spectrum measured for the sample containing the ^{15}N-labelled NAS/unlabelled NBS complex. Each assigned peak is labeled with the amino acid type and the residue number.

B

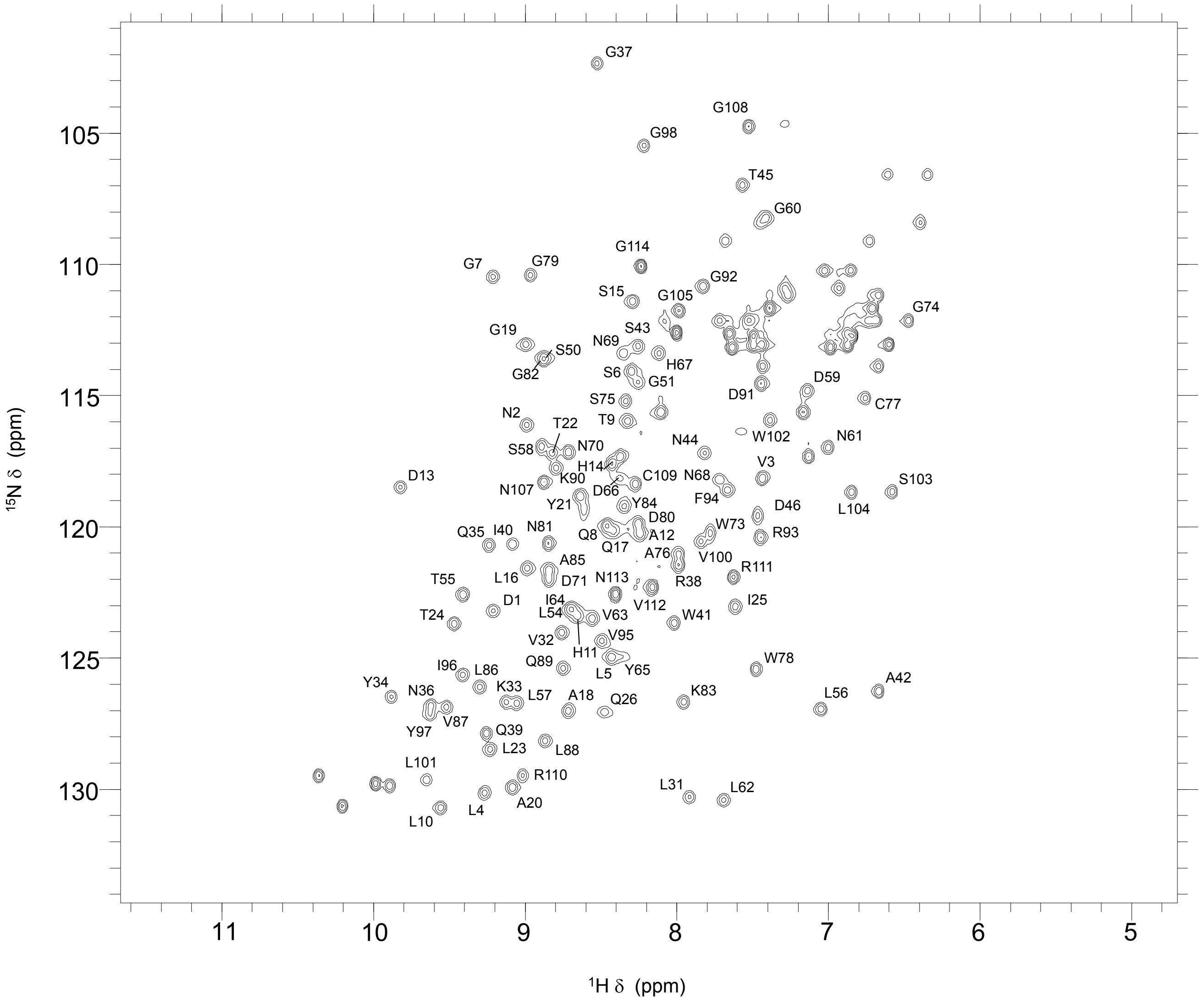

Figure S1 B ^{1}H-^{15}N HSQC spectrum measured for the sample containing the unlabelled NAS/^{15}N-labelled NBS complex. Each assigned peak is labeled with the amino acid type and the residue number.

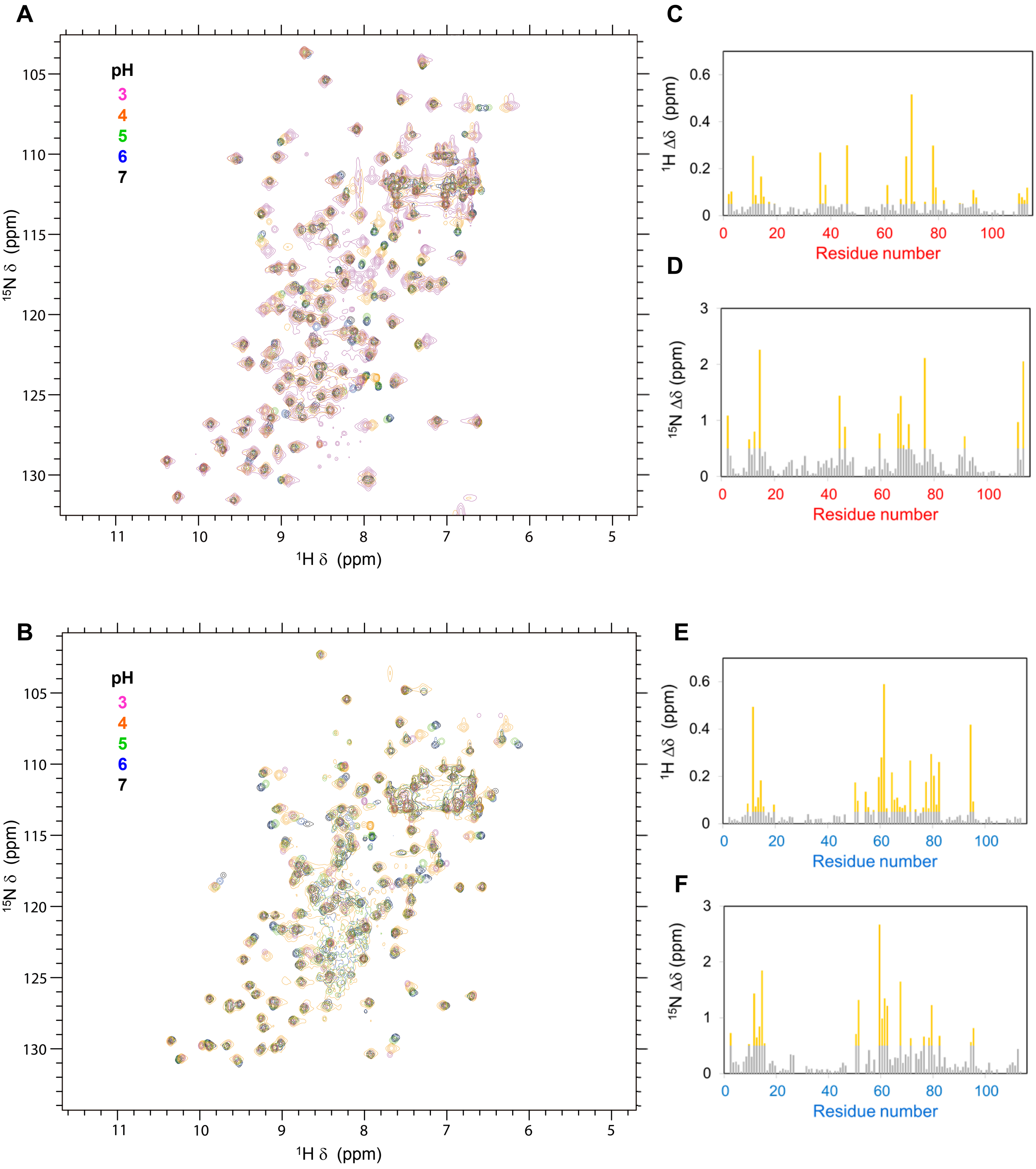

Figure S2 ^{1}H-^{15}N chemical shift changes following pH titration analysis. A-B) Superposition of ^{1}H-^{15}N HSQC spectra of the neoculin acidic subunit (A) and neoculin basic subunit (B). The spectra coloured in magenta, orange, green, blue, and black were acquired at pH 3.0, 4.0, 5.0, 6.0, and 7.0, respectively. All experiments were performed at 37° C. C-F) Bar charts showing ^{1}H, ^{15}N chemical shift changes in the backbone hydrogen of NAS (C) and NBS (E) and the backbone amides of NAS (D) and NBS (F) on the ^{1}H-^{15}N HSQC NMR spectra between pH 3 and 7. Parts of the bars corresponding to more than 0.05 ppm in the ^{1}H chemical shift or 0.5 ppm in the ^{15}N chemical shift are coloured in orange. The residues of NAS and NBS are depicted in red and blue letters, respectively.

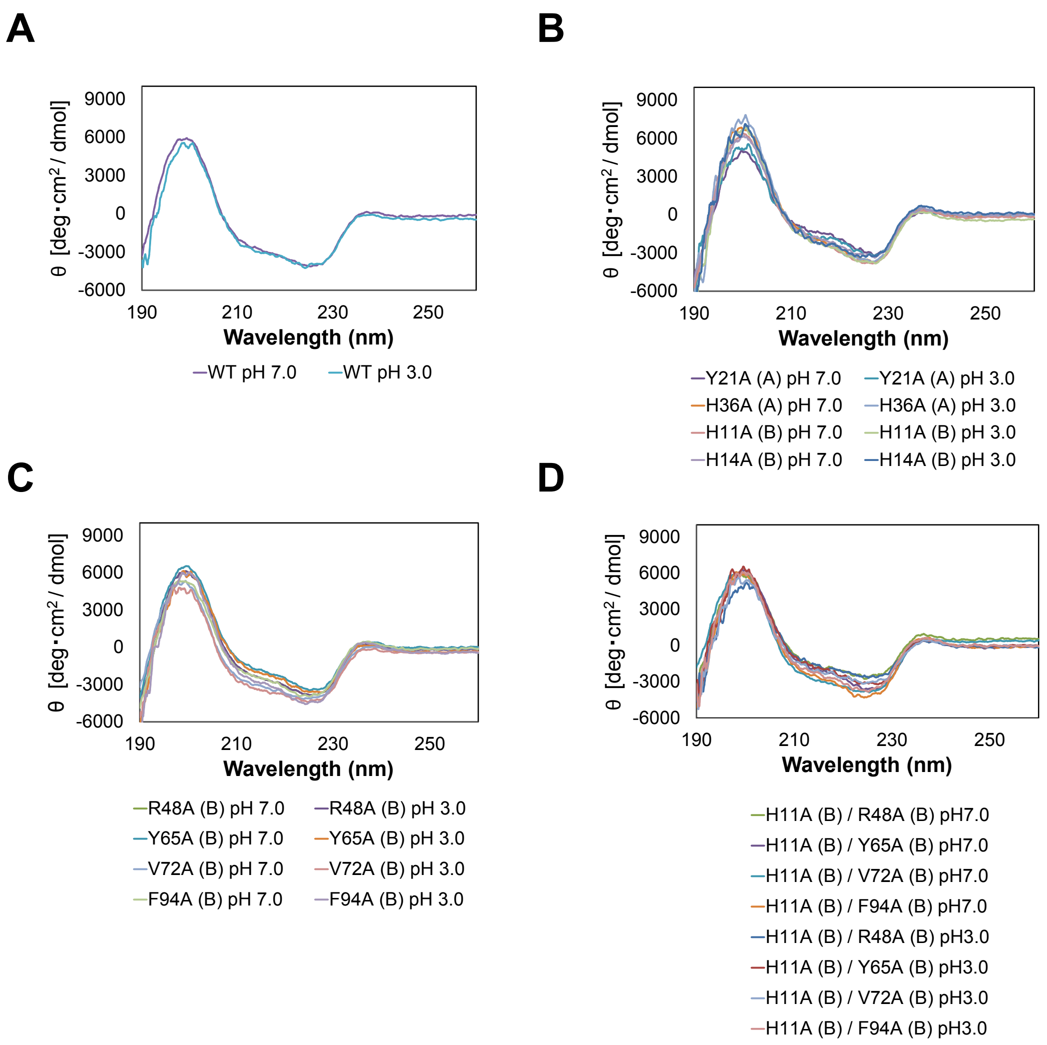

Figure S3 Far-UV CD spectra of the NCL mutants. A) wild type; B) NCL mutants that showed entirely pH-independent activity (NBS H11A) or slightly pH-dependent activities (NAS Y21A, H36A, and NBS H14A); C) NCL mutants that showed changes in their agonist and antagonist potencies; D) NCL double-mutants. The spectra were recorded in 20 mM sodium phosphate buffer, pH 7.0 or 20 mM sodium citrate buffer, pH 3.0.
